# ZnO nanoparticle induced electrical modulation and charge storage in *Aloe vera* leaves

**DOI:** 10.1039/d6na00129g

**Published:** 2026-04-20

**Authors:** Kajal Gautam, Mohit Bhatt, Archna Sagdeo, Anil Kumar Sinha

**Affiliations:** a Department of Chemistry, School of Advanced Engineering, UPES Dehradun 248007 India gautamkajal1210@gmail.com; b Department of Physics, School of Advanced Engineering, UPES Dehradun 248007 India mhtt.mb@gmail.com anilksinha11@gmail.com; c Accelerator Physics & Synchrotron Utilisation Division, RRCAT Indore MP-452013 India; d HBNI, Training School Complex Anushakti Nagar Mumbai-248006 India

## Abstract

This study investigates the influence of zinc oxide (ZnO) nanoparticles on the electrical transport and charge storage behavior of living *Aloe vera* leaves. Defect-rich ZnO nanoparticles (∼25.5 nm, wurtzite structure) were introduced *via* root-mediated exposure at concentrations of 1, 5, and 10 mg L^−1^. Electrochemical impedance spectroscopy (20 Hz to 5 MHz) revealed a concentration-dependent modulation of the electrical response of leaf tissues. The phase angle increased from ∼65° (control) to ∼83° (10 mg L^−1^), indicating enhanced capacitive behavior and interfacial polarization. Equivalent circuit analysis showed a significant rise in grain boundary resistance (∼100 Ω to >10 k Ω), suggesting restricted ionic transport across intercellular interfaces. Concurrently, grain and grain boundary capacitances decreased by nearly two orders of magnitude, indicating reduced polarization and charge storage. Dielectric analysis showed suppressed permittivity and energy dissipation with increasing ZnO concentration. AC conductivity results revealed a transition from long-range ionic conduction (*s* ≈ 0.0066) to localized hopping transport (*s* ≈ 0.98). Electric modulus analysis confirmed non-Debye relaxation behavior in treated samples. These findings demonstrate that ZnO uptake significantly alters the internal electrical properties of *Aloe vera* in a concentration-dependent manner, highlighting the potential of plant–nanomaterial systems for bioelectrical and sensing applications.

## Abstract 2

Root-mediated uptake of ZnO nanoparticles modulates the bioelectrical properties of living *Aloe vera* leaves. Impedance and dielectric analyses reveal suppressed ionic transport and localized hopping conduction. (Created with biorender.com)

## Graphical abstract

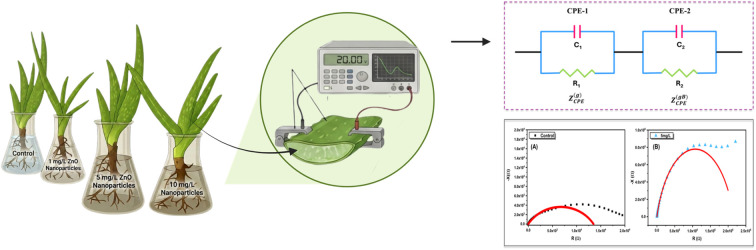

## 1. Introduction

Harnessing the intrinsic electrical properties of living plants offers a novel route toward developing self-sustaining energy-storage and signal-transduction systems. Plant tissues possess hydrated cellular frameworks and ion-conducting pathways that naturally facilitate charge transport and polarization,^1^ making them ideal candidates for exploring bio-ionic energy mechanisms. Such living architectures not only sustain continuous ionic flow through vascular bundles but also store electrochemical energy through capacitive and redox processes occurring at cellular interfaces.^2,3^ The intrinsic coupling between ionic movement and electrical potential in plants provides a unique opportunity to bridge biological systems with electronic materials for applications in bioenergy harvesting, soft bioelectronics, and sustainable power interfaces.^4^ The hydrated cellular matrix of plants consists of electrolytic sap,^5^ membrane channels,^6^ and conductive vascular tissues that promote polarization under an external electric field.^7^ These biological structures inherently act as distributed capacitors, where the balance between resistive and capacitive elements determines overall energy-storage efficiency. Integrating engineered nanomaterials into these ionic frameworks can further modulate their dielectric and electrochemical behavior by altering charge mobility, interfacial polarization, and dipolar relaxation processes.^8^ Among different nanomaterials, metal oxides are particularly effective in tuning electrochemical properties because of their high surface activity,^9^ defect-driven conductivity,^10^ and compatibility with hydrated environments.^11^

Zinc oxide (ZnO) nanoparticles are a versatile class of functional materials characterized by a wide bandgap (∼3.7 eV),^12^ high carrier mobility,^13^ and tunable surface chemistry.^14^ Their amphoteric nature and surface hydroxyl groups enable strong electrostatic interactions with polar molecules and ionic species present in biological fluids.^15^ In conventional energy-storage systems, ZnO-based materials are known to exhibit excellent pseudocapacitive behavior and fast charge-transfer kinetics due to their defect-mediated redox activity.^12,16^ When interfaced with biological matrices, ZnO nanoparticles can influence local ion distribution, enhance polarization at cell wall interfaces, and potentially induce dielectric modulation.^17,18^ Previous studies have reported that ZnO nanoparticles alter physiological parameters,^19,20^ photosynthetic activity,^21,22^ and enzymatic redox balance in several plant species;^23^ however, their direct impact on the electrical and dielectric characteristics of living plant tissues remains largely unexplored.

Electrochemical impedance spectroscopy (EIS) offers a non-destructive and quantitative means of probing such complex electrochemical interactions.^24^ By analysing the frequency-dependent response of a plant–nanomaterial system, one can distinguish between ionic transport in the cellular interior (grain) and interfacial polarization at membrane boundaries (grain boundary).^3,8^ Equivalent-circuit modeling further enables extraction of resistive, capacitive, and constant-phase parameters that describe charge transport, accumulation, and relaxation. Few studies have examined the impedance characteristics of succulent plants, where energy-storage behavior and hydration-related electrical responses have been observed in the presence of nanomaterials.^3,8^ In this work, *Aloe vera* was selected as a model plant owing to its high-water content, stable ionic conductivity, and well-defined gel-like leaf tissue,^25^ which collectively make it an ideal biological capacitor. The plant roots were immersed in ZnO nanoparticle suspensions at controlled concentrations (1, 5, and 10 mg L^−1^) to facilitate nanoparticle uptake through the vascular channels. Electrochemical impedance spectroscopy was employed to examine how ZnO incorporation modulates charge transport and relaxation dynamics within the plant leaves. Equivalent-circuit analysis, along with frequency-dependent evaluation of dielectric and modulus parameters, was used to gain insights into polarization strength, energy dissipation, and ionic mobility. To the best of our knowledge, this study provides the first systematic impedance-based investigation of ZnO nanoparticle induced electrical modulation in living *Aloe vera* leaves, demonstrating that concentration-dependent electrical responses can encode internal physicochemical changes within plant tissues.

## 2. Experimental section

### 2.1 Synthesis of ZnO nanoparticles

**Fig. 1:**
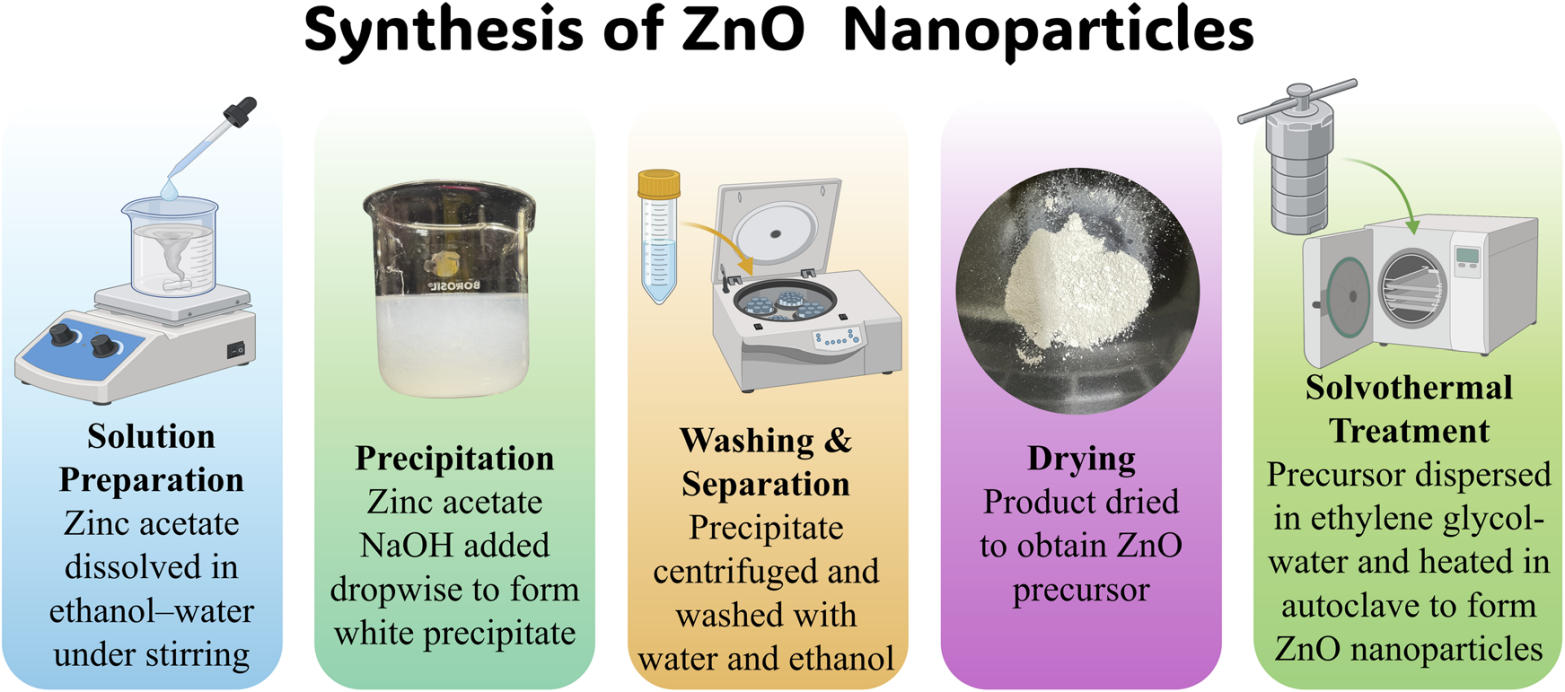
Schematic illustration of the synthesis of ZnO nanoparticles *via* a precipitation-solvothermal route (Created with https://www.biorender.com/).

Zinc oxide nanoparticles were synthesized using a modified precipitation–solvothermal approach with zinc acetate dihydrate as the precursor. In a typical procedure, 1.372 g of Zn(CH_3_COO)_2_·2H_2_O was dissolved in a 1 : 1 (v/v) ethanol–water mixture (250 mL total) under vigorous stirring to obtain a clear solution. A freshly prepared 0.10 M NaOH solution (125 mL) was then added dropwise, leading to the immediate formation of a white precipitate. The reaction mixture was stirred further to ensure complete precipitation, after which the solid product was collected by centrifugation and washed repeatedly with deionized water until the pH of the supernatant approached neutrality. The washed precipitate was rinsed with ethanol to remove residual organics and dried at 220 °C for 6 h to obtain the ZnO precursor. For the solvothermal treatment, the dried precursor was dispersed in a mixture of ethylene glycol (50 mL) and deionized water (5 mL), preheated to 80 °C, and stirred to achieve uniform dispersion. The suspension was transferred into a Teflon-lined stainless-steel autoclave and maintained at 200 °C for 6 h. After natural cooling, the resulting product was separated, thoroughly washed with deionized water and acetone to eliminate glycol residues, and dried at 80 °C to yield phase-pure ZnO nanoparticles (Fig. 1).

### 2.2 Plant treatment and sample preparation

**Fig. 2:**
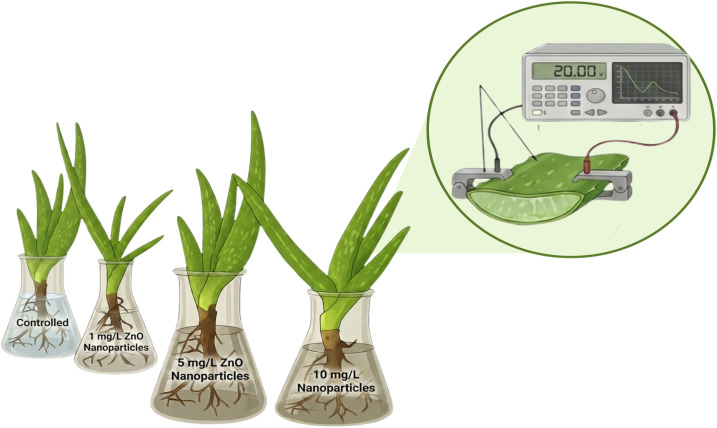
Schematic illustration of *Aloe vera* plants exposed to ZnO nanoparticle suspensions at different concentrations (control, 1, 5, and 10 mg L^−1^) through root immersion. The call-out shows the electrochemical impedance measurement of an *Aloe vera* leaf using an impedance analyzer, where the leaf section is placed between the sample holder to record frequency-dependent electrical response (Created with https://www.biorender.com/).

Healthy *Aloe vera* plants of uniform age and physiological condition were procured from a local nursery in Dehradun, India, and divided into four groups: one control and three treated sets exposed to ZnO nanoparticle suspensions of 1, 5, and 10 mg L^−1^, respectively (Fig. 2). Before treatment, roots were gently uprooted and washed several times with deionized water to remove soil particles and surface impurities. The cleaned plants were then immersed in 100 mL of their respective ZnO suspensions and maintained under ambient laboratory conditions (temperature 25 ± 2 °C) for one week to facilitate root-mediated uptake of nanoparticles. Root-mediated uptake was employed to ensure controlled and uniform internalization of ZnO nanoparticles within the plant system. This approach facilitates efficient transport through vascular tissues and provides a reproducible pathway for studying internal electrical and electrochemical responses. In contrast, foliar application methods may result in non-uniform deposition and limited penetration due to surface barriers.^26^ Following exposure, the plants were reintroduced into soil and allowed to stabilize under natural growth conditions to regain physiological balance, after which impedance measurements were performed on *Aloe vera* leaf sections of 1 × 1 cm^2^ cross section.

### 2.3 Characterization

The structural, optical, and electrochemical characterization studies of the synthesized ZnO nanoparticles and treated *Aloe vera* leaves were performed using standard analytical techniques.

#### 2.3.1 X-ray diffraction (XRD)

The crystalline structure of ZnO nanoparticles was examined using a D8 ADVANCE ECO diffractometer (Bruker, Germany) with Cu Kα radiation (*λ* = 1.5406 Å), operated at 40 kV and 40 mA. Diffraction patterns were recorded over a 2*θ* range of 10°–80° with a step size of 0.02° and analyzed to confirm phase purity, lattice parameters, and crystalline orientation.

#### 2.3.2 Fourier transform infrared spectroscopy (FTIR)

FTIR spectra were collected on a PerkinElmer Frontier spectrometer over the 500–4000 cm^−1^ range to identify surface functional groups and Zn–O vibrational features. ZnO powder was mixed with spectroscopic-grade KBr and pressed into pellets. The spectra were used to assess surface hydroxylation and oxygenated species relevant to nanoparticle dispersion and interfacial activity.

#### 2.3.3 Photoluminescence (PL) spectroscopy

Optical emission spectra were obtained using a PerkinElmer LS 45 fluorescence spectrometer. Samples dispersed in ethanol were excited at 365 nm. The spectra provided information on electronic transitions and defect-related emissions associated with oxygen vacancies and zinc interstitials.

#### 2.3.4 Electrochemical impedance spectroscopy (EIS)

Electrical measurements on *Aloe vera* leaves, both control and ZnO treated, were performed using a Wayne Kerr 6500B impedance analyzer (RRCAT, Indore, India) over a frequency range of 20 Hz to 5 MHz. Leaf sections (1 × 1 cm^2^) were rinsed with deionized water, mounted in the sample holder, and analyzed under ambient conditions. The impedance data were used to extract resistive, capacitive, and constant-phase parameters through equivalent circuit modeling, providing insight into charge transport and energy storage behavior.

## 3. Results and discussion

### 3.1 Structural and optical Characterization of ZnO nanoparticles

**Fig. 3:**
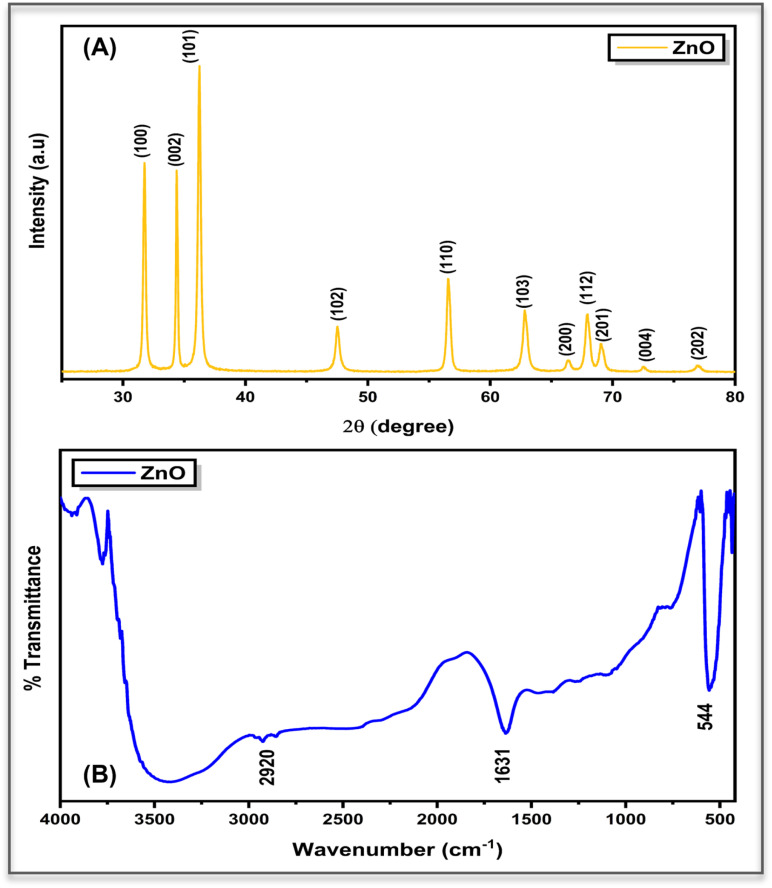
(A) XRD pattern showing the crystalline nature of ZnO nanoparticles and (B) FTIR spectrum indicating the presence of characteristic functional groups and Zn–O vibrations.

X-ray diffraction (XRD) patterns of the synthesized ZnO nanoparticles display reflections indexed to the hexagonal wurtzite phase (space group *P*6_3_*mc*; JCPDS 36-1451), confirming phase purity and crystallinity.^12^ The calculated lattice constants (*a* = *b* = 3.25 Å, *c* = 5.21 Å) and the average crystallite size (∼25.5 nm) derived from Debye–Scherrer and Williamson–Hall analyses verify nanoscale dimensions with a microstrain of 0.0023 ± 0.00059.^12^ The corresponding dislocation density (1.54 × 10^−3^ nm^−2^) evidences the presence of lattice distortions and defect sites.^16^ These nanoscale features and defect centres are expected to play a decisive role in interfacial charge transport by facilitating ion exchange and enhancing the electrochemical reactivity of ZnO in hydrated biological environments (refer Fig. 3(A)).

The FTIR spectrum of the synthesized ZnO nanoparticles reveals distinct absorption bands associated with surface functional groups and Zn–O lattice vibrations (refer Fig. 3(B)). A broad absorption band in the region of 3300–3500 cm^−1^ corresponds to O–H stretching vibrations, indicating the presence of surface hydroxyl groups, most likely arising from adsorbed moisture.^27^ These hydroxyl groups improve the hydrophilic nature of the nanoparticles and support their stable dispersion in aqueous media, which in turn aids their interaction with plant roots and enhances uptake. A weak band observed near 2920 cm^−1^ is attributed to C–H stretching vibrations, possibly originating from trace organic remnants from the synthesis process.^28^ The absorption band at ∼1631 cm^−1^ is attributed to the asymmetric stretching vibration of carboxylate (–COO^−^) groups coordinated to surface Zn^2+^ ions, indicating the presence of surface-bound zinc carboxylate or carbonate-derived oxygen-containing species on chemically prepared ZnO nanoparticles.^29^ Such surface features contribute to colloidal stability in water and may influence the interaction of nanoparticles with root surfaces, thereby affecting internal ion transport pathways within the plant system. In the lower wavenumber region, the prominent band below 544 cm^−1^ is characteristic of Zn–O stretching vibrations, confirming the formation of crystalline ZnO.^30^ Altogether, the FTIR results suggest that the nanoparticles possess surface functionalities that promote aqueous dispersibility and support effective root-mediated uptake, which is important for assessing their concentration-dependent impact on plant impedance behaviour.

**Fig. 4:**
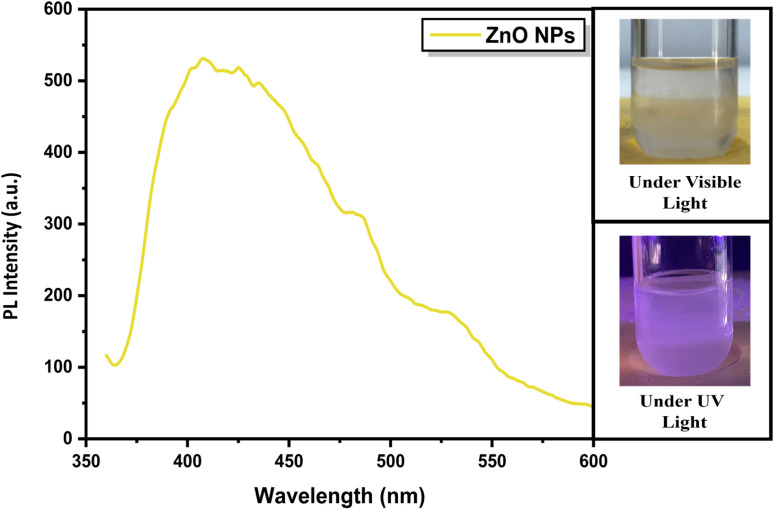
Photoluminescence spectrum of ZnO nanoparticles showing a broad visible emission band centered around ∼440 nm. Insets show the ZnO nanoparticle dispersion under normal light (milky appearance) and bluish-white emission under UV excitation (365 nm).

Photoluminescence analysis was carried out to further examine the defect-related optical behaviour of the synthesized ZnO nanoparticles. The dispersion appeared milky white under normal light, which is characteristic of nanosized particles causing strong light scattering in the medium. When excited under UV radiation (365 nm), the same suspension exhibited a distinct bluish-white emission (refer Fig. 4), indicating active radiative transitions within the ZnO lattice.^31^

The PL spectrum shows a broad visible emission band centered around ∼440 nm, which is commonly associated with intrinsic defect states such as oxygen vacancies and zinc interstitials.^32^ These defects create localized energy levels within the bandgap that facilitate radiative recombination, producing emission in the blue–green region (∼440–500 nm). The presence of this broad and intense visible emission suggests a high density of surface and lattice defect sites in the synthesized nanoparticles.^33^ Such defect-mediated luminescence is typical of colloidal ZnO nanostructures and supports their ability to participate in charge trapping and release processes, which is relevant to their interaction with hydrated biological systems.

### 3.2 Impedance analysis and equivalent circuit modelling

**Fig. 5:**
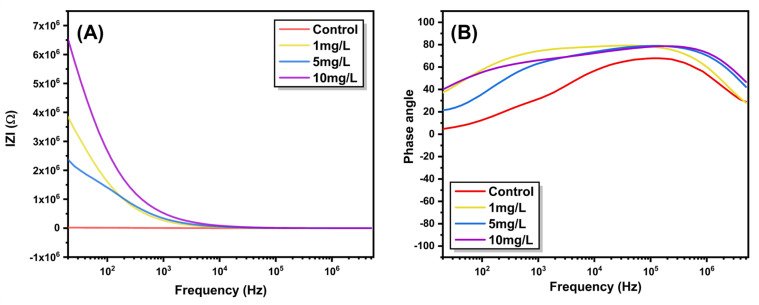
Bode plots showing (A) impedance magnitude (|*Z*|) and (B) phase angle of control and ZnO-treated *Aloe vera* leaves measured in the 20 Hz–5 MHz frequency range. The decrease in |*Z*| and the rise in the phase angle with increasing ZnO concentration indicate enhanced ionic transport and a transition toward capacitive behavior within plant tissues.

Electrochemical impedance spectroscopy (EIS) provides deep insight into charge transport, dielectric relaxation, and interfacial polarization mechanisms within biological systems.^34^ In the present study, the impedance spectra of *Aloe vera* leaves treated with different concentrations of ZnO nanoparticles were recorded in the frequency range of 20 Hz to 5 MHz at room temperature. The real (*Z*′) and imaginary (*Z*″) components of impedance are related to resistive and capacitive elements of the tissue, respectively. The overall impedance of the system is expressed as follows^35^:

1 *Z*(*ω*) = *Z*′(*ω*) + *jZ*″(*ω*)

where *Z*′and *Z*″denote the real and imaginary components and *ω* = 2π*f*is the angular frequency. Fig. 5(A) depicts the frequency dependence of the impedance magnitude, calculated from:2
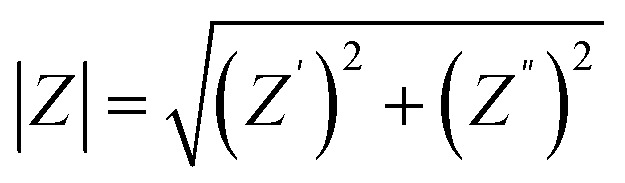


At low frequencies, high |*Z*| values arise from interfacial polarization and limited charge mobility, while at higher frequencies, ions can respond faster to the alternating field, reducing |*Z*|.^36^ The untreated leaf exhibits the lowest impedance due to unimpeded ionic motion through the hydrated cellular matrix. Increasing ZnO concentration results in a notable rise in |*Z*|, particularly at lower frequencies, confirming that nanoparticles restrict ion transport by partially blocking vascular channels and modifying interfacial charge density. The phase-angle spectra (Fig. 5(B)) provide complementary information about relaxation behavior. The phase angle, defined as follows^37^

3 *θ* = tan^−1^(*X*/*R*)

**Fig. 6:**
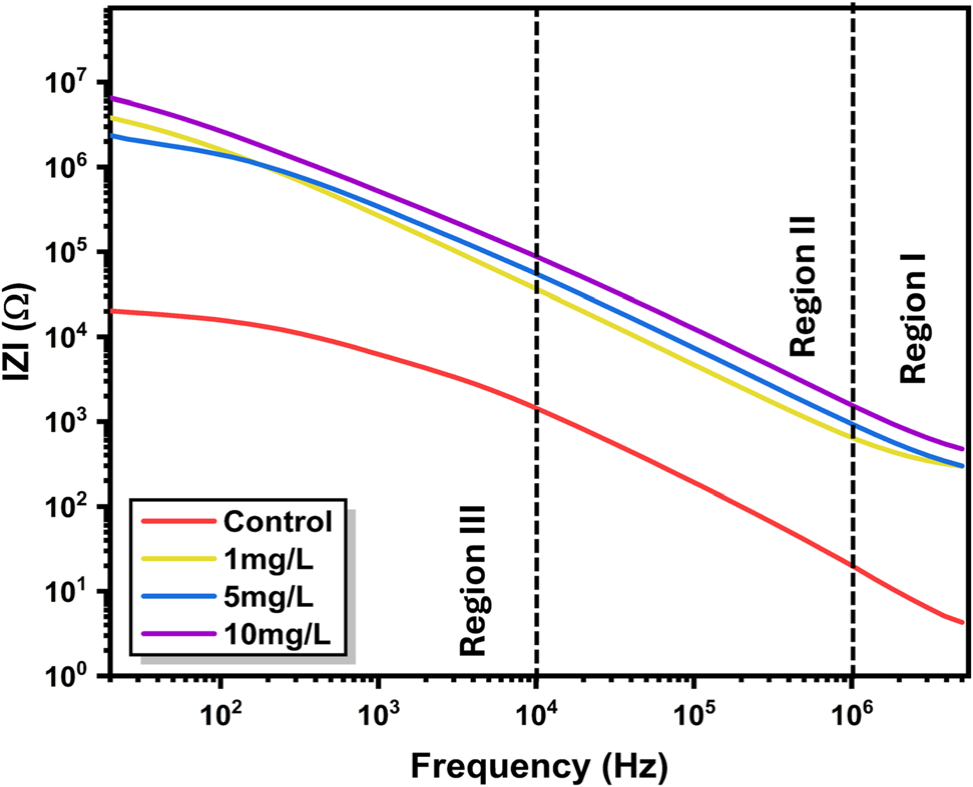
Log–log plot of impedance magnitude (|*Z*|) as a function of frequency for control and ZnO-treated *Aloe vera* leaves. The marked regions denote distinct conduction regimes associated with bulk (Region III), interfacial or membrane-related processes (Region II), and electrode polarization effects (Region I).

**Fig. 7:**
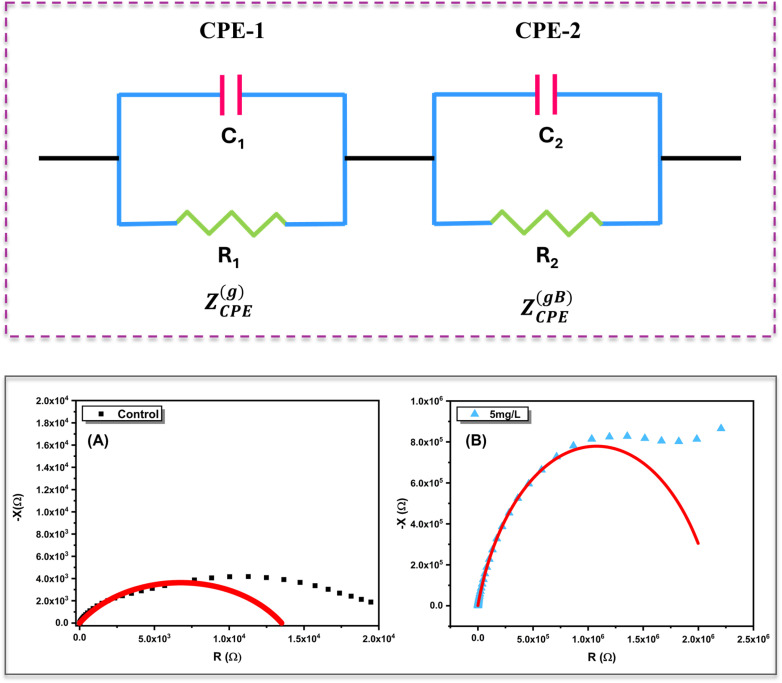
(a) Equivalent circuit model used to analyse the impedance response of *Aloe vera* leaves, representing grain (bulk) and grain boundary contributions with corresponding resistive and constant phase elements. (b) Nyquist plots (*Z*′–*Z*″) of *Aloe vera* leaves under (A) control and (B) ZnO nanoparticle-treated (5 mg L^−1^) conditions.

This parameter reflects the phase difference between the applied voltage and current response and provides insight into the relative contributions of resistive and capacitive elements within the system. The phase-angle spectra (Fig. 5(B)) exhibit a pronounced shift with nanoparticle exposure, rising from ∼65° in the control to ∼83° at 10 mg L^−1^ ZnO. Such an increase signifies a transition from resistive to dominantly capacitive response, reflecting improved dipolar orientation and reduced interfacial charge leakage. The approach toward a near-ideal capacitive phase at intermediate frequencies denotes strong polarization of intracellular electrolytes and enhanced ion confinement within hydrated parenchyma tissues. These effects originate from ZnO induced modification of cell wall interfaces, which increase the local electric-field gradient and facilitate charge separation across membrane domains. Fig. 6 shows the logarithmic |*Z*|-frequency plot which divides the electrical response into three domains. Region I (high frequency) represents bulk or intracellular conduction; Region II corresponds to the cell membrane and intercellular boundaries; Region III (low frequency) reflects electrode and space-charge polarization. A consistent upward shift of |*Z*| across all regions after nanoparticle exposure demonstrates that ZnO affects both bulk and interfacial conduction pathways. The reduced slope at higher frequencies indicates enhanced dielectric stability, where polarization processes become less dispersive under the applied field. Notably, each ZnO concentration produces a distinct and reproducible impedance signature across the investigated frequency range, enabling clear electrical discrimination between treatment levels. After analysing the frequency-dependent behavior, the impedance data were further interpreted using an equivalent circuit model to understand the contributions from different conduction regions within the plant tissue. The selected model effectively captures both the bulk (cellular) and interfacial (membrane) responses and consists of a bulk resistance (*R*_g_) with a constant-phase element (CPE-1) connected in series with a grain-boundary resistance (*R*_gb_) and another constant-phase element (CPE-2) (refer Fig. 7). The use of CPEs instead of ideal capacitors accounts for the non-Debye relaxation behavior typically observed in biological matrices, where ionic conduction and dipolar polarization are spatially distributed.^3^

**Fig. 8:**
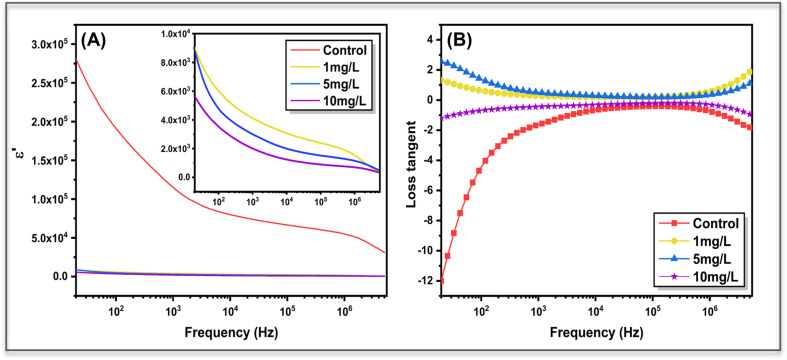
Frequency-dependent dielectric behavior of control and ZnO-treated *Aloe vera* leaves showing (A) real part of dielectric permittivity (*ε*′) and (B) loss tangent (tan *δ*). The insets highlight the responses of the 1, 5, and 10 mg L^−1^ ZnO-treated samples for improved clarity.

To further validate the impedance analysis and assess the applicability of the equivalent circuit model, Nyquist plots (*Z*′–*Z*″/*R*–*X*) along with the corresponding fitted curves have been included (Fig. 8). The plots present both the experimental data points and the fitted arcs, showing good agreement between the measured response and the modelled impedance behavior.

For the control sample, a depressed semicircular arc is observed in the high- to mid-frequency region, indicating the contribution of intrinsic electrical processes within the plant tissue. The non-ideal nature of the arc suggests a distribution of relaxation times, which is typical of biologically heterogeneous systems. Upon ZnO nanoparticle treatment, the arc diameter increases noticeably along the real axis, reflecting an increase in impedance and a modification in charge transport behavior within the *Aloe vera* tissue.

The impedance of a CPE is expressed as:

4 *Z*_CPE_ = *A*^−1^_CPE_*jω*^−*n*^

which can be resolved into its resistive and capacitive components as:4a
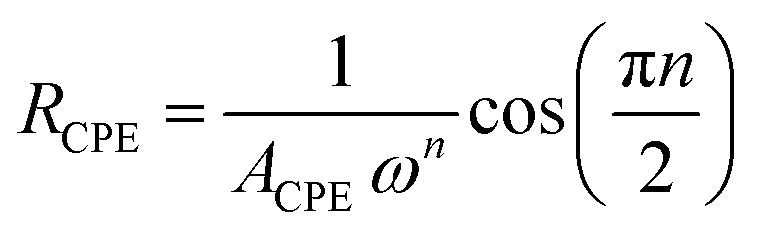
4b
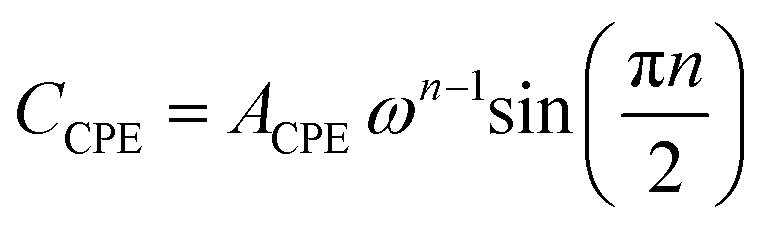


Using relations 4a and 4b, the grain and grain boundary parameters listed in Table 1 were evaluated from the fitted *A*_CPE_ and *n* values. The characteristic onset frequency of 2.5 MHz was used for calculating the grain (intracellular) resistance and capacitance, whereas a frequency of 20 kHz was taken for determining the grain boundary (intercellular) parameters. The characteristic frequencies used for evaluating grain and grain boundary parameters were selected based on the frequency regions where bulk and interfacial processes dominate. The high-frequency region corresponds to intracellular (grain) conduction, where the influence of electrode polarization is minimal, while the intermediate frequency region represents grain boundary contributions associated with cell membranes and intercellular interfaces. These frequencies were selected from regions showing stable impedance behavior to ensure reliable extraction of equivalent circuit parameters^3,24,37^

**Table 1:** Grain and grain boundary resistance, CPE equivalent circuit parameters

Sample	(g) *A*_CPE_ (F s^*n*−1^)	*n*_1_	(gB) *A*_CPE_ (F s^*n*−1^)	*n*_2_	(g) *C*_CPE_ (F)	(gB) *C*_CPE_ (F)	(g) *R*_CPE_ (Ω)	(gB) *R*_CPE_ (Ω)
Control	9.06 × 10^−6^	0.55	2.09 × 10^−8^	0.93	6.89 × 10^−9^	9.27 × 10^−9^	7.90	100.5
1 mg L^−1^	4.22 × 10^−6^	0.38	1.52 × 10^−9^	0.88	2.59 × 10^−10^	3.79 × 10^−10^	361.4	4042
5 mg L^−1^	4.97 × 10^−8^	0.64	8.71 × 10^−10^	0.89	1.51 × 10^−10^	2.43 × 10^−10^	267.5	5888
10 mg L^−1^	1.83 × 10^−8^	0.68	5.91 × 10^−10^	0.88	1.04 × 10^−10^	1.47 × 10^−10^	334.7	10 104

Both grain resistance (*R*_g_) and grain boundary resistance (*R*_gb_) increased systematically with increasing ZnO concentration, indicating a progressive restriction in charge transport within the leaf tissue. The value of *R*_g_ increased significantly from 7.90 Ω in the control sample to 361.4 Ω at 1 mg L^−1^, followed by 267.5 Ω at 5 mg L^−1^ and 334.7 Ω at 10 mg L^−1^, suggesting that nanoparticle uptake alters ionic movement within the intracellular regions. A much stronger variation was observed in *R*_gb_, which rose sharply from ∼100.5 Ω in the control to 4042 Ω, 5888 Ω, and 10 104 Ω at 1, 5, and 10 mg L^−1^, respectively. This pronounced increase indicates that ZnO nanoparticles significantly hinder charge transport across intercellular junctions, likely through interactions with cell wall polysaccharides and membrane-associated proteins. In contrast, the capacitances of both grain (*C*_g_) and grain boundary (*C*_gb_) regions decreased notably after ZnO incorporation, dropping from 6.89 × 10^−9^ F and 9.27 × 10^−9^ F in the control to the order of 10^−10^ F at higher concentrations. This reduction in capacitance reflects a diminished ability of *Aloe vera* tissues to store charge, which may result from partial blocking of ion channels and suppression of water-mediated polarization. Since capacitance in plant tissues is closely associated with water content and dipolar relaxation of mobile species, the observed decline suggests that ZnO nanoparticles reduce the effective dielectric response while simultaneously increasing resistive barriers within both bulk and interfacial regions.

### 3.3 Frequency dependent dielectric behavior

The dielectric response of *Aloe vera* leaves treated with different concentrations of ZnO nanoparticles was investigated by analysing the frequency dependence of the real part of the dielectric constant (*ε*′) and the dielectric loss tangent (tan *δ*), as shown in Fig. 8. For the control sample, *ε*′ exhibits a very high value in the low-frequency region, followed by a gradual decline with increasing frequency. This behaviour is typical of hydrated biological tissues, where interfacial polarization, space-charge accumulation, and electrode polarization dominate the low-frequency regime. As the applied frequency increases, these slow polarization mechanisms are progressively suppressed, resulting in a reduced dielectric constant governed by faster dipolar relaxation processes. In contrast, ZnO-treated samples show a marked reduction in *ε*′ across the entire frequency range, with the magnitude decreasing systematically as nanoparticle concentration increases. This concentration-dependent suppression of the dielectric constant indicates that ZnO incorporation restricts long-range ionic displacement and reduces charge accumulation at cellular and intercellular interfaces (refer Fig. 8(A)). The diminished polarization strength suggests that nanoparticle uptake modifies the internal electrical landscape of the leaf matrix, likely by altering ionic pathways and membrane-associated polarization sites. At higher frequencies, *ε*′ values for all samples converge, reflecting the dominance of intrinsic dipolar polarization, which is less sensitive to nanoparticle concentration.

The dielectric loss behaviour, represented by the loss tangent (tan *δ*), provides further insight into energy dissipation within the system (Fig. 8(B)). The untreated leaf displays a strongly negative tan *δ* at low frequencies, indicative of significant dielectric losses associated with electrode polarization and extensive ionic motion. Upon ZnO treatment, the magnitude of tan *δ* decreases and shifts toward less negative or near-zero values, particularly in the mid-frequency region, signifying reduced dielectric losses and more constrained charge dynamics. Each treated sample exhibits a distinct tan *δ* profile, highlighting concentration-dependent changes in relaxation behaviour induced by nanoparticle–tissue interactions. At higher frequencies, tan *δ* values tend to stabilize for all samples, indicating that dielectric losses are governed primarily by localized dipolar relaxation rather than long-range ionic conduction. Overall, the systematic modification of both *ε*′ and tan *δ* with ZnO concentration demonstrates that nanoparticle uptake alters the balance between polarization strength and energy dissipation within *Aloe vera* tissues. Importantly, the concentration-dependent modification of the dielectric constant and loss behaviour indicates that ZnO uptake produces electrically distinguishable polarization states within the plant matrix, reinforcing the use of impedance-derived dielectric parameters for discriminating nanoparticle exposure levels.

### 3.4 AC conductivity

**Fig. 9:**
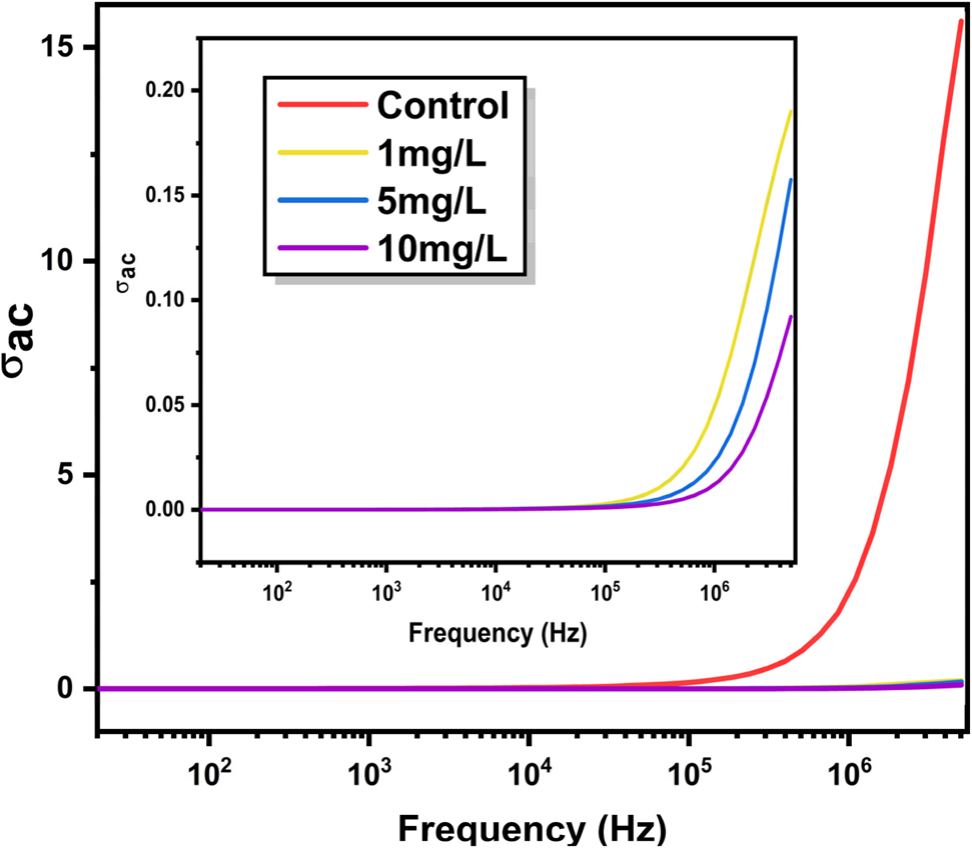
Frequency-dependent AC conductivity (*σ*_ac_) of control and ZnO-treated *Aloe vera* leaves. The transition from frequency-independent to dispersive behaviour indicates the coexistence of dc conduction and hopping-dominated transport mechanisms influenced by nanoparticle concentration.

The frequency dependence of AC conductivity (*σ*_ac_) for control and ZnO-treated *Aloe vera* leaves is shown in Fig. 9. For all samples, *σ*_ac_ increases with frequency, indicating dispersive charge transport behaviour typical of ionically conducting biological systems. However, the magnitude and dispersion profile of *σ*_ac_ vary significantly with ZnO nanoparticle concentration, reflecting concentration-dependent modification of charge transport pathways within the plant tissue.

In the low-frequency region, *σ*_ac_ exhibits a weak frequency dependence, particularly for the ZnO-treated samples. This regime corresponds to long-range ionic conduction through hydrated intracellular and vascular channels, where charge motion is governed primarily by DC-like transport. The control sample shows comparatively higher *σ*_ac_ values in this region, suggesting relatively unhindered ionic mobility in the absence of nanoparticle incorporation. In contrast, ZnO treated samples display suppressed *σ*_ac_, indicating restricted ion transport arising from nanoparticle–membrane interactions and increased structural barriers within the tissue matrix, consistent with the increased resistive components extracted from impedance analysis. At higher frequencies, *σ*_ac_ increases sharply for all samples, marking the transition to localized charge transport dominated by hopping mechanisms. To better understand the high-frequency behavior, an inset has been included in Fig. 9. The inset reveals a clear concentration-dependent separation of *σ*_ac_ at higher frequencies, where the control sample exhibits the highest conductivity, followed by 1 mg L^−1^, 5 mg L^−1^, and 10 mg L^−1^. This trend indicates that increasing ZnO concentration suppresses charge transport, which can be attributed to enhanced charge carrier confinement and increased resistive barriers within the plant matrix.

The DC conductivity (*σ*_dc_) for each sample was obtained by extrapolating the low-frequency plateau of the *σ*_ac_*versus* frequency curves, where the conductivity becomes weakly dependent on frequency.^3^ To quantitatively analyse this dispersive behaviour, the AC conductivity data were fitted using Jonscher's universal power law:^37^

5 *σ*_ac_(*ω*) = *σ*_dc_ + *Aω*^*s*^

where, *σ*_dc_ represents the frequency-independent DC conductivity, *A* is a pre-exponential factor related to the density of localized states, *ω* is the angular frequency, and *s* is the frequency exponent that reflects the nature of charge transport.

**Fig. 10:**
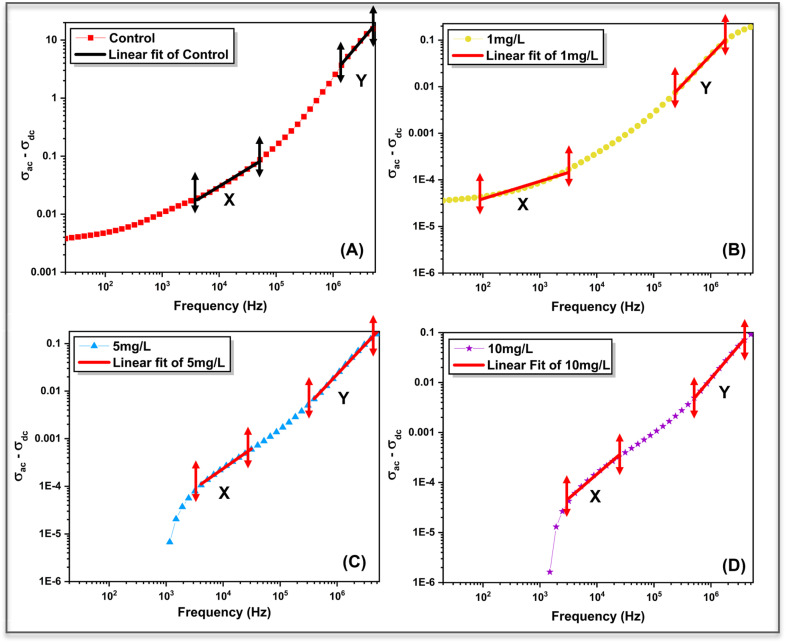
Log–log plots of *σ*_ac_–*σ*_dc_*versus* frequency for (A) control, (B) 1 mg L^−1^, (C) 5 mg L^−1^, and (D) 10 mg L^−1^ ZnO-treated samples. Solid lines represent linear fits based on Jonscher's universal power law, used to extract grain and grain-boundary conduction parameters.

The *σ*_ac_–*σ*_dc_*versus* frequency plots (Fig. 10(A–D)) reveal two distinct linear regions, labelled X and Y, corresponding to grain (bulk) and grain boundary conduction, respectively. In the grain-dominated region (X), charge transport is governed by localized hopping within intracellular domains, whereas in the grain boundary region (Y), conduction is influenced by intercellular interfaces and membrane-associated barriers. The extracted Jonscher parameters for both regions are summarized in Table .2. For the control sample, the grain conduction exponent (*S*_1_ = 0.0066) is extremely low, indicating DC-like ionic transport through extended pathways. Upon ZnO treatment, *s*_1_ increases progressively from 0.375 (1 mg L^−1^) to 0.98 (10 mg L^−1^), signifying a gradual transition from long-range ionic conduction to strongly localized hopping transport. This trend demonstrates that increasing nanoparticle concentration introduces disorder and spatial confinement, limiting extended ionic motion within the plant matrix.

**Table 2:** Jonscher's parameters of AC conduction in grain and grain boundary regions

Sample	Grain conduction			Grain boundary conduction		
	*S*_1_	*A*_1_ (S.s^*n*^)	*R* ^2^	*S*_2_	*A*_2_ (S.s^*n*^)	*R* ^2^
Control	0.0066	4.8 × 10^−5^	0.92	1.78	1.4 × 10^−13^	0.997
1 mg L^−1^	0.375	6.95 × 10^−5^	0.961	1.27	1.02 × 10^−9^	0.998
5 mg L^−1^	0.837	1.04 × 10^−7^	0.995	1.27	5.18 × 10^−10^	0.995
10 mg L^−1^	0.98	1.75 × 10^−8^	0.995	1.345	1.02 × 10^−10^	0.999

In the grain boundary region, the conduction exponent (*S*_2_) remains greater than unity for all samples, indicating highly dispersive transport dominated by interfacial polarization and constrained charge relaxation at cellular boundaries. The slight increase in S_2_ with ZnO concentration suggests enhanced barrier-controlled transport arising from nanoparticle accumulation at membrane and intercellular interfaces. The corresponding reduction in the pre-exponential factor (*A*_2_) with increasing ZnO concentration further supports a decrease in available hopping sites and charge carrier mobility in the grain boundary regions. The systematic evolution of *σ*_ac_ behaviour and Jonscher parameters with ZnO concentration confirms that nanoparticle uptake progressively alters the dominant charge transport mechanism in *Aloe vera*, shifting it from extended ionic conduction toward localized hopping-dominated transport. These concentration-dependent transport signatures, when considered alongside the impedance and dielectric analyses, demonstrate that the electrical response of the plant tissue encodes variations in nanoparticle exposure through measurable and distinguishable conductivity characteristics.

### 3.5 Electric modulus

**Fig. 11:**
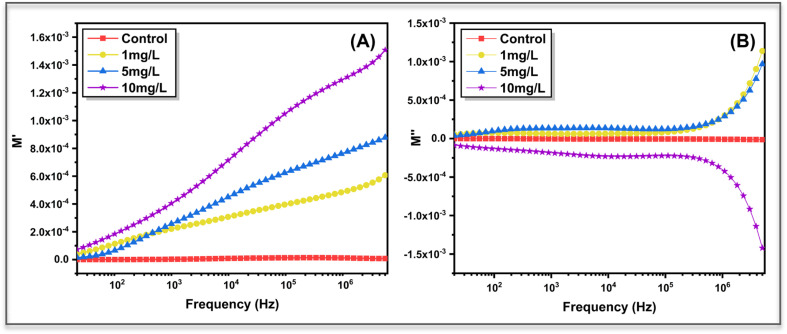
Frequency dependence of the (A) real part (*M*′) and (B) imaginary part (*M*″) of the electric modulus for control and ZnO-treated *Aloe vera* leaves at different nanoparticle concentrations. The concentration-dependent evolution of *M*′ and the dispersive nature of *M*″ reflect modified bulk relaxation and non-Debye charge dynamics induced by ZnO incorporation.

To further examine bulk relaxation processes while minimizing electrode polarization effects, electric modulus analysis was performed using the relations as follows:

6 M* = 1/ε* = *M*′ + *jM*″

where M*is the complex electric modulus, ε*is the complex permittivity, *M*′and *M*″are the real and imaginary parts of the modulus, and *j*is the imaginary unit. Fig. 11(A) shows the variation of the real part of the electric modulus (*M*′) with frequency. The control sample exhibits negligible *M*′ values across the frequency range, indicating dominant electrode polarization and weak bulk relaxation. In contrast, ZnO treated samples show a clear, concentration-dependent increase in *M*′ with frequency, suggesting suppression of long-range polarization and enhanced contribution from bulk relaxation mechanisms. The progressive rise in *M*′ with increasing ZnO concentration reflects restricted ionic mobility and increased resistive character within the plant matrix, consistent with impedance and AC conductivity results.

The imaginary part of the modulus (*M*″), shown in Fig. 11(B), provides insight into relaxation dynamics. The untreated leaf displays minimal *M*″ response, indicating the absence of a well-defined bulk relaxation process. Upon ZnO treatment, dispersive *M*″ behaviour emerges, with magnitude and sign strongly dependent on nanoparticle concentration. The treated samples exhibit broadened relaxation features rather than sharp peaks; indicative of non-Debye relaxation associated with structural heterogeneity and distributed charge dynamics in the biological tissue. No distinct relaxation maximum is observed within the measured frequency window, suggesting that relaxation processes are either highly dispersed or occur outside the experimental range.

**Fig. 12:**
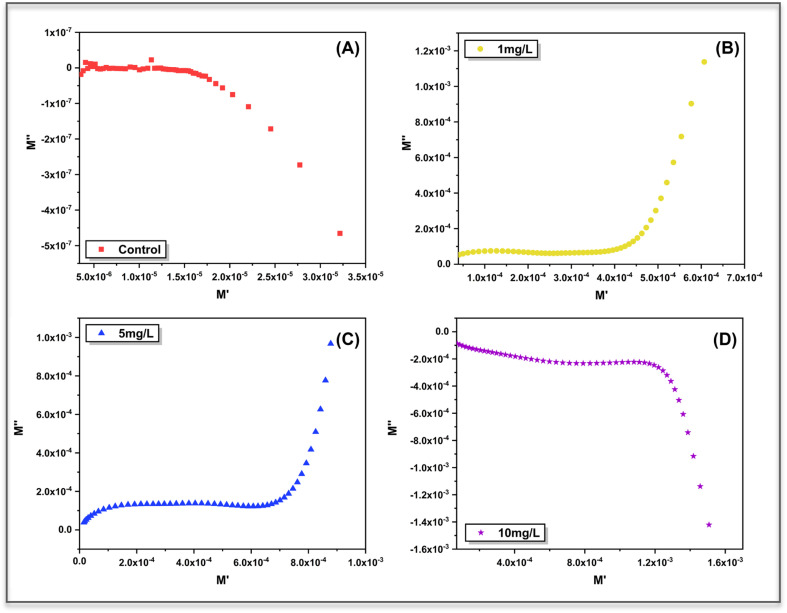
Complex electric modulus plots (*M*″ *versus M*′) for (A) control and ZnO treated *Aloe vera* leaves at (B) 1 mg L^−1^, (C) 5 mg L^−1^, and (D) 10 mg L^−1^ ZnO concentrations. The distorted, non-semicircular trajectories indicate distributed relaxation behavior and the absence of a single Debye-type relaxation process, reflecting heterogeneous charge dynamics within the plant tissue.

The relaxation behaviour was further visualized using *M*′–*M*″ plots (Fig. 12(A–D)), which provide a compact representation of bulk charge dynamics independent of electrode polarization. The control sample exhibits a highly compressed and featureless trajectory, consistent with the negligible *M*′ and *M*″ responses observed in the frequency domain and confirming the absence of a dominant bulk relaxation process.

In contrast, ZnO treated samples show the development of asymmetric, open trajectories whose shape and extent evolve systematically with nanoparticle concentration. At lower concentrations (1 mg and 5 mg L^−1^), the gradual curvature in the *M*′–*M*″ plane reflects the emergence of short-range ionic relaxation within the tissue matrix, while the lack of a closed semicircle indicates non-Debye behaviour arising from distributed relaxation times. At higher ZnO loading (10 mg L^−1^), the trajectory shifts markedly toward negative *M*″ values at higher *M*′, suggesting enhanced charge carrier confinement and suppressed relaxation strength. The appearance of negative *M*″ values at higher nanoparticle concentration can be attributed to enhanced interfacial polarization and charge trapping^38^ within the heterogeneous plant matrix. The increased resistive barriers and restricted ionic mobility lead to delayed relaxation processes, resulting in anomalous and dispersive modulus behavior. Such features are characteristic of non-Debye relaxation in structurally disordered systems and are often observed under conditions of strong interfacial effects.^39^ This evolution corroborates the dispersive *M*″ behaviour observed in the frequency domain and confirms that ZnO incorporation progressively modifies bulk relaxation pathways rather than introducing a single dominant relaxation mode.

## 4. Conclusion

This study provides a comprehensive understanding of how ZnO nanoparticle uptake modulates electrical transport, dielectric polarization, and relaxation dynamics in living *Aloe vera* leaves. Structural and optical characterization confirmed the successful synthesis of defect-rich ZnO nanoparticles with nanoscale dimensions, whose surface hydroxylation and intrinsic defects facilitate effective interaction with hydrated plant tissues. Root-mediated exposure to ZnO nanoparticles resulted in a clear and concentration-dependent alteration of the electrical response of the leaves. Impedance analysis revealed a substantial increase in both grain and grain boundary resistances, with grain boundary resistance rising from ∼100 Ω in untreated samples to more than 10 k Ω at the highest nanoparticle concentration, indicating significant hindrance to ionic transport across intercellular interfaces. Simultaneously, the pronounced reduction in grain and grain boundary capacitances reflects a diminished polarization strength and reduced charge storage capability within the hydrated biological matrix. Dielectric and AC conductivity analyses further demonstrated suppression of long-range ionic displacement and space-charge polarization, accompanied by a gradual transition from extended ionic conduction to localized hopping-dominated transport as nanoparticle concentration increased. The evolution of the grain conduction exponent toward unity and the emergence of distributed, non-Debye relaxation behavior in electric modulus analysis confirm increased heterogeneity and confinement of charge carriers within the tissue. Importantly, each level of ZnO exposure produces a distinct and reproducible electrical signature, underscoring the sensitivity of plant tissues to internal physicochemical modifications induced by nanomaterial uptake. Overall, these findings establish that living plant systems can function as responsive bioelectrical platforms, where impedance-derived parameters encode internal transport and polarization changes. This work advances the understanding of plant-nanomaterial interactions and opens new possibilities for utilizing plant tissues in bio-sensing, soft bioelectronics, and sustainable bio-energy-related applications.

## Author contributions

K. G.: project administration, wrote the original manuscript, performed the synthesis and characterization experiments, and conducted complete data analysis. M. B.: reviewed and edited the manuscript, provided key suggestions related to the chemistry of the ZnO material, and assisted in the analysis of XRD, FTIR, and impedance data. A. S.: reviewed and edited the manuscript and provided essential resources. A. K. S.: project administration, supervision, reviewed and edited the manuscript, and assisted in data analysis.

## Conflicts of interest

The authors declare that there are no conflicts of interest regarding the publication of this paper.

## Data Availability

The datasets generated during and/or analysed during the current study are not publicly available due to their inclusion in the author's ongoing PhD work but are available from the authors upon reasonable request.
